# The Diagnosis of Cervical Dysplasia in a University Hospital Using Pap Smear and Colposcopy in the Western Region of Saudi Arabia: A Correlational Study

**DOI:** 10.7759/cureus.23242

**Published:** 2022-03-17

**Authors:** Wejdan o Baamer, Nisreen Anfinan, Maram Sait, Omar Baghlaf, Nashwa AlDardir, Asem Sebghatallah, Bayan Alkhalili, Mohammed Sulaimani, Rahaf Alghamdi, Khalid H Sait

**Affiliations:** 1 Obstetrics and Gynaecology, King Abdulaziz University Faculty of Medicine, Jeddah, SAU; 2 Internal Medicine, King Abdulaziz University Faculty of Medicine, Jeddah, SAU; 3 Family Medicine, Faculty of Medicine, Ibin Sina University, Jeddah, SAU

**Keywords:** histopathology, correlations studies, pap smear, colposcopy, cervical dysplasia

## Abstract

Objectives

To assess the diagnostic performance of Pap smear screening with or without human papillomavirus (HPV) testing and colposcopy in detecting preinvasive lesions of the cervix among women with reference to histopathological findings.

Materials and methods

We performed a retrospective study in a tertiary care center of the clinical and pathological records of women with evocative symptomatology. The diagnostic performance of Pap smear screening and colposcopy was analyzed. The sensitivity and specificity of Pap smear screening and colposcopy in detecting preinvasive lesions of the cervix were calculated in 388 patients.

Results

The mean age was 45.12 years, and the most frequent gynecological symptoms included abnormal bleeding (17.2%) and postcoital bleeding (10.9%). Histopathology showed abnormal results in 26.5% of the 388 patients, including cervical intraepithelial neoplasia 1 (CIN 1; 20.4%), CIN 2 (2.8%), CIN 3 (1.3%), and SCC (1.3%). Both Pap smear screening and colposcopy were highly sensitive in detecting CIN 1+ (94.2%vs.93.2%, respectively) and CIN 2+ (100.0% vs.95.8%, respectively) intraepithelial lesions; however, Pap smears had very low specificity in detecting both CIN 1+ (8.1% vs.73.7%, respectively) and CIN 2+ (8.0% vs. 59.3%, respectively) compared with colposcopy. When combined with HPV status, the specificity of Pap smear increased considerably.

Conclusion

It has become a high priority to improve the efficiency of cervical cancer (CC) screening programs by optimizing the practice of Pap smear screening, increasing the test specificity, and implementing systematic cytology-HPV co-testing.

## Introduction

According to various international sources, cervical cancer (CC) ranked as the fourth most prevalent cancer among women worldwide in 2018, with an estimated age-standardized incidence of 13.1 per 100,000 women and large interregional variability [1,2]. CC was responsible for over 311 million deaths worldwide during the same year, accounting for the fourth leading cause of mortality among women, with the highest mortality rates occurring in medium-and lower-resource countries [2].

Persistent human papillomavirus (HPV) infections constitute the strongest risk factor for CC, notably those caused by high-risk types of HPV, such as types 16 and 18, which have a high oncogenic potential [3,4]. Therefore, the early detection of high-risk HPV infections, combined with screening for preinvasive lesions, notably intraepithelial neoplasia grade 2, cervical intraepithelial neoplasia 2 (CIN 2), and CIN 3, represents the universally admitted paradigm in CC prevention to date, in addition to prophylactic vaccination for HPV [4,5].

Most of the noninvasive screening programs involve a Papanicolaou smear (Pap smear), which is carried out by cytological collection from the transformation zone of the cervix. The specimen undergoes specific straining and microscopic analysis to detect the presence and grade of eventual abnormal cytological findings, which are classified from negative for intraepithelial lesion and malignancy (NILM) to squamous cell carcinoma (SCC) using the Bethesda system [6]. However, because the sensitivity of the Pap smear test is questionable, it is increasingly recommended that it be combined with HPV screening and genotyping to improve the sensitivity [5-7]. In Saudi Arabia, according to the recommendations issued by a Saudi expert panel in 2016, all women with a risk of CC should benefit from HPV DNA testing followed by colposcopy, or Pap smear testing followed by colposcopy, to screen for CIN 2 and higher-grade lesions [8].

Screening programs have been estimated to decrease the incidence of CC and the associated mortality by up to 88% and 98%, respectively [9]. However, in developing countries, the lack of sufficient facilities and trained staff to perform the procedures and interpret the findings has impeded the implementation and efficacy of CC screening [10]. In the Middle East region, and more specifically in Saudi Arabia, recent reports showed inadequate awareness levels about CC and screening programs, which was associated with very low attendance rates for screening programs among target populations [11]. The low coverage of screening among the target population had previously resulted in a high percentage of cases diagnosed at advanced stages that required chemoradiation therapy [12].

From the perspective of optimizing the currently established screening efforts, it is of paramount importance to study the effectiveness of the screening methods used to improve the detection rates. Thus, the present study was primarily designed to assess the diagnostic performance of Pap smear screening and colposcopy in detecting preinvasive and invasive malignant lesions of the cervix among women with evocative symptomatology by reference to histopathological findings. Additionally, they investigated HPV screening by estimating the screening rate and analyzing the correlation of HPV status combined with Pap smear results with histological findings. Second, the prevalence of CC was estimated, and the associated demographic and clinical factors were analyzed. To the best of our knowledge, this is the first study to locally evaluate the cytohistological correlation and discrepancy of conventional Pap smear testing with histopathology findings.

## Materials and methods

Study design

This retrospective study was conducted in a tertiary care center from February 13, 2010, to December 31, 2019. We reviewed the clinical and pathological records of Pap smear, HPV, colposcopy, and cervix biopsy histopathological findings, which were carried out regarding the screening for cervical cancer among women with evocative symptomatology.

Inclusion and exclusion criteria

We included all Pap smears that were performed on sexually active women who were aged 21-65 years and who presented to the Gynecology Department during the study period with any of the following evocative symptoms: abnormal vaginal discharge, abdominal pain, irregular menstrual bleeding, postmenopausal bleeding, postcoital bleeding, intermenstrual bleeding, or prolapse. Pap smears performed on asymptomatic women aged older than 65 years or younger than 21 years and those performed on women who were already diagnosed with cervical cancer, pregnant women, and post-total hysterectomy patients were excluded, as well as smears with an unsatisfactory quality for evaluation.

Diagnostic testing protocol

Pap smear specimens were taken prior to colposcopy using liquid-based cytology, and high-risk HPV testing was used upon availability in women older than 30 years of age, while cervical biopsies were taken for suspicious areas found on colposcopy. The cytological interpretation of the smears was made according to the Bethesda system 2014, which categorizes the findings into the following classifications: NILM; atypical squamous cell of undetermined significance (ASCUS); atypical squamous cell cannot exclude high-grade squamous intraepithelial lesion (ASC-H); low-grade squamous intraepithelial lesion (LSIL); high-grade squamous intraepithelial lesion (HSIL); atypical glandular cells favor neoplastic (AGC-N); atypical glandular cells not otherwise specified (AGC-NOS); and SCC [13].

Colposcopy abnormalities were indicated by the presence of acetowhite areas and abnormal vessels, and findings were categorized as normal, CIN 1, and CIN 2-3. In cases of the identification of abnormalities on colposcopy, biopsy specimens were taken from suspect areas and sent to pathology. The results were categorized as normal, CIN 1, CIN 2, CIN 3, squamous cell carcinoma (SCC), and adenocarcinoma, in accordance with the WHO classification [14].

Data collection

Data were collected using an Excel sheet, which was divided into two sections: (1) demographic and clinical data, including age, parity, symptoms (abnormal bleeding, postcoital bleeding, vaginal discharge, etc.), oral contraceptive use, smoking status, and personal history of dysplasia; and (2) HPV status, pap smear, colposcopy, and histopathological findings.

Statistical analysis

Statistical analysis was performed with the Statistical Package for Social Sciences version 21.0 for Windows (SPSS Inc., Chicago, IL, USA). Categorical variables are presented as frequencies and percentages, while continuous variables are presented as the mean ± standard deviation (SD). The diagnostic performance of Pap smear screening and colposcopy was analyzed by dichotomizing the respective findings into normal and abnormal findings, where abnormal findings were defined as ASCUS or higher grade for Pap smear screening and abnormal vessels or CIN for colposcopy. Using histopathological findings as the gold standard, the sensitivity, specificity, and positive and negative predictive values of Pap smear screening and colposcopy were calculated to detect two grades of abnormalities, including CIN 1 or higher-grade abnormalities (sensitivity analysis 1) and CIN 2 or higher-grade abnormalities (sensitivity analysis 2). Furthermore, the proportional agreement of Pap smear findings (positive or negative) with colposcopy and histopathology was calculated. Factors associated with CC as indicated by positive histopathology (CIN 1+) were analyzed using the chi-square test, Fisher’s exact test, the independent t-test, or the Mann-Whitney U test, as appropriate, depending on the type and distribution of the factor variable. A p-value of <0.05 was considered to reject the null hypothesis.

Ethical approval

The Unit of Biomedical Human Ethics Research Committee for KAUH approved the study protocol based on the international recommendations on human subject research and according to the principles of the Helsinki declaration (Reference 532-18). The requirement for informed consent was waived by the ethical committee.

## Results

Participant characteristics

During the study period, the Pap smear and colposcopy findings of 448 eligible women were reviewed, of which 388 led to biopsies being performed based on the colposcopy findings. The demographic and clinical data of the 448 patients are presented in Table 1. These showed a mean (SD) age = 45.12 (9.86) years, high parity (57.1% had four or more children), and a low HPV positivity rate (21.7%), while 37.1% of the patients had an unknown HPV status. The most frequent gynecological symptoms included abnormal bleeding (17.2%), postcoital bleeding (10.9%), and postmenopausal bleeding (10.7%).

**Table 1:** Participants’ demographic and clinical characteristics (N=448) Results are frequency (percentage), except if otherwise specified. SD: standard deviation; P75: 75th centile; HPV: human papillomavirus; OCP: oral contraception; ^§^more than one symptom may be reported in one patient.

Parameter	Category	Frequency	Percentage
Age (years)	Mean, SD (range=21, 75)	45.12	9.86
Parity	Median, P75 (range=0, 12)	4	6
	Nulliparous	26	5.8
	1-3	122	27.2
	4-5	129	28.8
	6+	127	28.3
	Not reported	44	9.8
HPV status	Negative	185	41.3
	Positive	97	21.7
	Unknown	166	37.1
Symptoms^§^	Abnormal bleeding	77	17.2
	Postcoital bleeding	49	10.9
	Postmenopausal bleeding	48	10.7
	Intermenstrual bleeding	6	1.3
	Vaginal discharge	1	0.2
OCP usage	No	212	47.3
	Yes	122	27.2
	Not specified	114	25.4
Smoking	No	272	60.7
	Yes	26	5.8
	Not specified	150	33.5
History of dysplasia	No	331	73.9
	Yes	117	26.1

**Figure 1:**
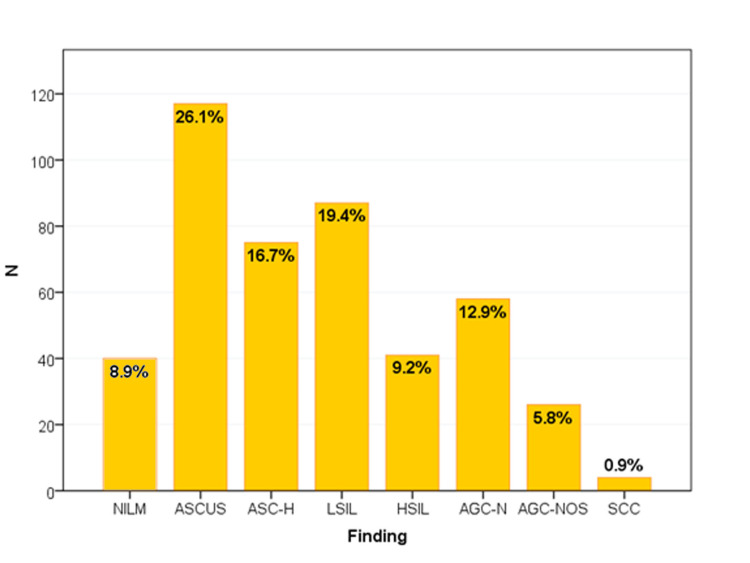
Papanicolaou smear interpretation according to the 2014 Bethesda system (N=448) NILM: Negative for intraepithelial lesion or malignancy; ASCUS: atypical squamous cell of undetermined significance; ASC-H: atypical squamous cell cannot exclude HSIL; LSIL: low-grade squamous intraepithelial lesion; HSIL: high-grade squamous intraepithelial lesion; AGC-N: atypical glandular cells favor neoplastic; AGC-NOS: atypical glandular cells not otherwise specified; SCC: squamous cell carcinoma

The majority of the participants had abnormal Pap smears (91.1%), with ASCUS (26.1%), LSIL (19.4%), and ASC-H (16.7%) accounting for the most frequent abnormalities (Figure 1).

On the other hand, only 38.6% of the participants had abnormal colposcopy, with CIN 1 being the most frequent abnormality (29.5%). Histopathology was performed for 388 participants and showed abnormal results in 26.5% of them, including CIN 1 (20.4%), CIN 2 (2.8%), CIN 3 (1.3%), and SCC (1.3%) (Table 2). Thus, based on the histopathological findings (N=388), the prevalence of abnormal cervical lesions was 26.5% (95% CI=22.2%, 31.2%) when considering all abnormal findings and 6.2% (95% CI=4.0%, 9.1%) when considering findings of CIN II or higher-grade lesions.

**Table 2:** Colposcopy and histopathology findings (N=448) Results are frequency (percentage), except if otherwise specified. CIN: cervical intraepithelial neoplasia; CIS: carcinoma in situ; SCC: squamous cell carcinoma; ADC: adenocarcinoma.

Investigation	Finding	No. cases	Percentage
Colposcopy (N=448)	Normal	275	61.4
	Abnormal vessels	5	1.1
	CIN 1	132	29.5
	CIN 2-3	36	8.0
Histopathology (N=388)	Normal	285	73.5
	CIN 1	79	20.4
	CIN 2	11	2.8
	CIN 3	6	1.6
	SCC	5	1.3
	ADC	2	0.5

By considering any abnormal cytological finding as a positive test result, Pap smear alone showed weak proportional agreement with colposcopy (44.9%), CIN 1 or higher-grade lesions in histopathology (30.9%), and CIN 2 or higher-grade lesions in histopathology (13.7%) (Table 3).

**Table 3:** Correlation of Pap smear with histopathology and colposcopy

Golden standard	PAP smear (test)			
	Negative	Positive	Total	Proportional agreement
Colposcopy				44.9%
Negative	34	241	275	
Positive	6	167	173	
Total	40	408	448	
Histopathology 1				30.9%
Negative	23	262	285	
Positive (CIN I or higher-grade)	6	97	103	
Total	29	359	388	
Histopathology 2				13.7%
Negative	29	335	364	
Positive (CIN II or higher-grade)	0	24	24	
Total	29	359	388	

Diagnostic performance analysis showed higher accuracy for colposcopy in detecting CIN 1+ and CIN 2+ intraepithelial lesions compared with Pap smear screening, including a high sensitivity for both colposcopy (93.2% and 95.8%, respectively) and Pap smear screening (94.2% and 100.0%, respectively) and a very low specificity of Pap smear screening (8.1% and 8.0%) compared with colposcopy (73.7% and 59.3%, respectively). When both screenings were combined, the performance did not differ significantly from that of colposcopy alone. When combined with HPV status, the specificity of Pap smear screening increased considerably, both in detecting CIN 1+ (77.7%) and CIN 2+ (50.0%); however, the sensitivity decreased to 41.6% and 73.3%, respectively (Table 4).

**Table 4:** Accuracy of colposcopy, Pap smear alone and combined with colposcopy and HPV status in detecting intraepithelial cervical neoplasia by reference to histopathology PPV: positive predictive value; NPV: negative predictive value; TP: true positive; TN: true negative; FP; false positive; FN: false negative; *combined Pap smear × HPV is considered positive when positive Pap smear and HPV are positive. Sensitivity=TP/TP+FN, Specificity=TN/TN+FP, PPV=TP/TP+FP, NPV=TN/TN+FN, accuracy=TP+TN/TP+TN+FP+FN.

Parameter	Golden standard (histopathology)	
	CIN 1	CIN 2
Colposcopy (N=388)		
Sensitivity	93.2% (86.5, 97.2%)	95.8% (78.9, 99.9%)
Specificity	73.7% (68.2, 78.7%)	59.3% (54.1, 64.4%)
PPV	56.1% (51.1, 61.0%)	13.5% (11.8, 15.3%)
NPV	96.8% (93.6, 98.4%)	99.5% (96.9, 99.9%)
Accuracy	78.9% (74.5, 82.8%)	61.6% (56.6, 66.5%)
Pap smear (N=388)		
Sensitivity	94.2% (87.8, 97.8%)	100.0% (85.8, 100.0%)
Specificity	8.1% (5.2, 11.9%)	8.0% (5.4, 11.2%)
PPV	27.0% (25.9, 28.2%)	6.7% (6.5, 6.9%)
NPV	79.3% (61.6%, 90.2%)	100.0% (-)
Accuracy	30.9% (26.4, 35.8%)	13.7% (10.4, 17.5%)
Pap smear × colposcopy* (N=388)		
Sensitivity	90.3% (82.9, 95.3%)	95.8% (78.9%, 99.9%)
Specificity	74.7% (96.3, 79.7%)	61.0% (55.8, 66.0%)
PPV	56.4% (51.2, 61.4%)	13.9% (12.2, 15.9%)
NPV	95.5% (92.2, 97.5%)	99.6% (97.0, 99.9%)
Accuracy	78.9% (74.5, 82.8%)	63.1% (58.1, 68.0%)
Pap smear × HPV* (N=243)		
Sensitivity	41.6% (30.4, 53.4%)	73.3% (44.9, 92.2%)
Specificity	77.7% (70.6, 83.8%)	50.0% (40.6, 59.4%)
PPV	46.4% (37.0, 56.1%)	15.9% (11.7, 21.3%)
NPV	74.1% (70.0, 77.9%)	93.6% (86.0, 97.2%)
Accuracy	66.3% (60.0, 72.2%)	52.7% (43.8, 61.5%)

To detect CIN 1+ lesions, positive HPV status was associated with the highest positivity rate both in cases in which the Pap smear was positive (46.4%) or negative (45.5%), while negative HPV status was associated with 25.0% and 14.3% positivity rates in cases in which the Pap smear was positive or negative, respectively (p=0.007). On the other hand, positive HPV status combined with a positive Pap smear was associated with 15.9% of CIN 2+ lesions detected, while a negative Pap smear was associated with no CIN 2+ lesions regardless of HPV status (Table 5).

**Table 5:** Correlation of PAP smear-HPV DNA combined results with histopathology findings (N=243) Test used: chi-square test; *statistically significant result (p<0.05).

Test results	N	Histopathology 1 (CIN 1+)		Histopathology 2 (CIN 2+)	
		Positivity rate	P-value	Positivity rate	P-value
HPV−/Pap−	7	14.3		0.0	
HPV+/Pap−	11	45.5		0.0	
HPV−/Pap+	156	25.0		2.6	
HPV+/Pap+	69	46.4	0.007*	15.9	0.001*

The detection of CIN 1 and higher-grade lesions in histopathology was associated with younger age (mean [SD]=43.86 [10.25] versus 46.13 [9.27] years, p=0.040) and lower parity (median 4 versus 5, p=0.029). Additionally, abnormal histopathology was more frequently detected in women who complained of abnormal bleeding (29.6% versus 12.9%, p=0.004) compared to their counterparts, as well as among women with known positive HPV status (46.3%) compared with those with negative (24.5%) and unknown (17.9%) status (p<0.001). Thus, a positive HPV status was associated with an OR of 3.94 (95% CI=2.14, 7.26) for the positive detection of cervical cancer in histopathology by reference to an unknown HPV status. No association of abnormal histopathology was found with the other symptoms, OCP usage, smoking, or history of dysplasia (Table 6).

**Table 6:** Demographic and clinical factors associated with abnormal cervix histopathology (N=388) Results are frequency (percentage), except if otherwise specified. SD: standard deviation; P75: 75th centile; HPV: human papilloma virus; OCP: oral contraception; *statistically significant association (p<0.05); test used: ^t^independent t-test, ^M^Mann-Whitney U test, ^F^Fisher’s exact test, otherwise chi-square test was used.

Factor	Category	Histopathology finding				
		Negative		Positive		p-value
		N	%	N	%	
Age (years)	Mean, SD	46.13	9.27	43.86	10.25	0.04*^t^
Parity	Median, P75	5	6	4	5	0.029*^M^
	Nulliparous	16	66.7	8	33.3	
	1-3	63	64.3	35	35.7	
	4-5	82	73.2	30	26.8	
	6+	94	81.0	22	19.0	0.045*
HPV status	Negative	123	75.5	40	24.5	
	Positive	43	53.8	37	46.3	
	Unknown	119	82.1	26	17.9	<0.001*
Abnormal bleeding	No	224	70.4	94	29.6	
	Yes	61	87.1	9	12.9	0.004*
Postcoital bleeding	No	255	73.5	92	26.5	
	Yes	30	73.5	11	26.8	0.965
Postmenopausal bleeding	No	252	72.8	94	27.2	
	Yes	33	78.6	9	21.4	0.426
Intermenstrual bleeding	No	282	73.6	101	26.4	
	Yes	3	60.0	2	40.0	0.612^F^
Vaginal discharge	No	284	73.4	103	26.6	
	Yes	1	100.0	0	0.0	1.000^F^
OCP usage	No	138	73.8	49	26.2	
	Yes	71	68.9	32	31.1	0.377
Smoking	No	172	72.6	65	27.4	
	Yes	13	61.9	8	38.1	0.298
History of dysplasia	No	214	73.3	78	26.7	
	Yes	71	74.0	25	26.0	0.897

## Discussion

This study examined the performance of Pap smear screening in detecting preinvasive disease of the cervix by comparison to coloscopy findings and by reference to the histopathological analysis of cervical biopsies collected from women with evocative symptoms, irrespective of their HPV status. Findings showed that both PAP smear screening and colposcopy were highly sensitive in detecting both CIN 1 or higher-grade (94.2% versus 93.2%, respectively) and CIN 2 or higher-grade (100.0% versus 95.8%, respectively) lesions; however, Pap smear screening had very poor specificity and PPV, which reduced the accuracy of the test to very low levels (<31%) compared to colposcopy (>61%). There was a weak proportional agreement (~45%) between colposcopy and Pap smear screening, and the combination of the two tests did not improve the performance compared to colposcopy alone, suggesting a poor added value of Pap smear screening. On the other hand, when combined with primary positive HPV status, Pap smear screening was more specific but less sensitive in detecting abnormal cervical lesions. Second, based on the histopathological analysis, the prevalence of invasive cervical lesions among the population was estimated at 6.2% when considering CIN II or higher-grade lesions, with a history of positive HPV being associated with a nearly fourfold risk and afflicted patients being relatively younger and having lower parity. Of note, more than one-third of the population had an unknown HPV status.

The use of PAP smear screening in our institution showed very high sensitivity both in detecting CIN 1 or higher-grade (94.2%) lesions and CIN 2 or higher-grade (100.0%) lesions. However, it showed very poor specificity (<10%) and PPV (<30%), which reduced the accuracy to very low levels (<31%). This considerably impacts the reliability of Pap smears as a screening test for cervical cancer. Furthermore, the comparison suggesting that colposcopy had better performance than Pap smear screening should be interpreted with caution, as the biopsy was guided by colposcopy findings in a number of patients per hospital protocol, which inevitably introduced a biasedly high specificity and overall accuracy of colposcopy.

Our findings are inconsistent with those from other authors reporting higher accuracy of Pap smear screening along with higher specificity (83-100%) and comparable (93-96%) or lower sensitivity (42-60%) using different definitions of positivity for Pap smear screening in detecting CIN2+ or higher-grade neoplasia [15-19]. Additionally, although studies comparing colposcopy and Pap smear screening reported discrepant conclusions regarding the performance of each test alone, the tests showed better performance and higher agreement in the present study, and their performance increased notably as the grade of the cytopathological result increased [16-18]. Several other studies suggested that colposcopy was more sensitive, while Pap smear screening was more specific [20,21].

This significant discrepancy between our findings and findings from the literature may further be explained by differences in the target population, as most of the other studies included asymptomatic women or patients enrolled in systematic screening programs, while in our study, only women with evocative symptoms were included, which may impact the interpretation of the Pap smear results and lead to the overdiagnosis of abnormalities. However, the previously cited study by Joshi et al. included a comparable population of symptomatic women and found that Pap smear screening was highly accurate (accuracy=80.0%), with a specificity of 95.8%, a PPV of 94.4%, and lower sensitivity (65.4%) than in our study [19]. Thus, we conclude that the higher sensitivity of Pap smear screening in our study, with the high false-positive rate, is likely to be due to an overinterpretation of the findings, which may question the technical performance and highlight the need to review the specimen collection and interpretation protocol. It must be noted that a high rate of false-positive results may have a non-negligible psychological impact on patients, as this may lead to distress, anxiety about developing cancer, or fear of death, irrespective of the knowledge and severity of Pap smear abnormalities [22]. This is another reason to make sure that the technical performance of the test is up to par. This will make the method more effective and the results more accurate.

The present study showed a history of positive HPV among only 21.7% of the participants, while 37.1% of the participants had unknown HPV status. In Saudi Arabia, the epidemiological picture of genital HPV infection is not well established because of the scarcity of population-based studies and the common belief that, as a sexually transmitted disease, HPV infection is not prevalent in conservative Saudi society [23]. The few available data showed HPV detection rates of less than 10% among healthy women [24,25], which is remarkably lower than the global figures. However, these rates increased to 32% in hospital-based samples [26] and to 43% in women with evocative symptomatology [27]. This is consistent with the present study showing an HPV positivity rate of 34.4% when considering only participants with known HPV status among symptomatic women. On the other hand, it must be emphasized that these relatively low figures should not limit the efforts of screening and research, as HPV causes 89% of local invasive cervical cancers, with types 16 and 18 representing approximately 80% of the positive samples [28]. In the present study, a history of positive HPV serology was associated with an OR of 3.94 (95% CI=2.14, 7.26) for the positive detection of cervical cancer in histopathology by reference to an unknown HPV status. This stresses the importance of promoting systematic screening among the general population by raising awareness about HPV infection transmission and related risks and alleviating the eventual psychological and social barriers.

Furthermore, local studies showed low levels of knowledge and awareness among Saudi females about the HPV vaccine and its role in preventing cervical cancer and other HPV-related diseases, in addition to relatively low percentages of acceptance to receive the vaccine [29]. Several sociodemographic factors for poor knowledge and reluctance to vaccinate were identified, which should be integrated into the design of targeted awareness campaigns.

The use of HPV status together with Pap smear findings was more specific and more accurate in detecting both CNI 1+ and CNI 2+ intraepithelial neoplasia than Pap smear screening alone; however, the sensitivity of the two tests declined considerably, which may reduce screening performance. However, the latter conclusion cannot be generalized because data on HPV status were collected from personal history, which is often based on a patient’s declaration, and analyzed without distinction between high-risk and low-risk serotypes. Nonetheless, it was demonstrated that cytology-HPV co-testing performs better than Pap smear screening alone by maximizing the sensitivity of detection for intraepithelial neoplasia without decreasing the specificity of the test. This led to a shift in the paradigm for CC screening, from cytology-based methods to hybrid screening strategies, including Pap smear screening and HPV testing, which are increasingly recommended as the reference methods for CC screening in developed countries [5,7,17,30]. In Saudi Arabia, the updated guidelines recommend using HPV DNA testing followed by colposcopy as the preferred screening method, while the next preferred method includes Pap smear screening followed by colposcopy [8]. Consequently, the implementation of HPV testing needs to be reconsidered in our center and other local institutions to improve the effectiveness of the screening program.

The limitation of this study is that it is a retrospective study that introduced bias in the inclusion of cases, namely, in the selection of patients for biopsy, which was guided by routine practice based on clinical and colposcopy findings. Additionally, the very high sensitivity of Pap smear screening compared with its very low accuracy suggests an overinterpretation of the findings.

The sensitivity and specificity analyses of the two tests hinder the generalizability of the findings. Additionally, the very high sensitivity of Pap smear screening contrasts with its very low accuracy, suggesting an overinterpretation of the findings and calling for a review of the collection and interpretation protocol.

## Conclusions

The use of PAP smear screening in our institution showed very high sensitivity both in detecting CIN 1 or higher-grade and CIN 2 or higher-grade lesions; however, it showed very poor specificity and PPV compared with that reported internationally, which reduced the reliability of its use as a screening test for CC. The introduction of systematic HPV testing combined with cytology should be effectively implemented to improve the efficiency of the screening program. Further prospective studies using a more appropriate design are warranted to assess the reliability of the different screening methods, notably co-testing strategies.
